# Fibrous Histiocytoma in the Far Lateral Frontal Sinus—A Rare Observation

**DOI:** 10.3390/diagnostics15010056

**Published:** 2024-12-28

**Authors:** Alexander Wilhelmer, Peter Kiss, Michael Habenbacher, Luka Brčić, Alexandros Andrianakis

**Affiliations:** 1 Department of Otorhinolaryngology, Medical University of Graz, Auenbruggerplatz 26, 8010 Graz, Austria; 2 Diagnostic and Research Institute of Pathology, Medical University of Graz, Auenbruggerplatz 26, 8010 Graz, Austria

**Keywords:** sinonasal tumor, sinonasal neoplasms, skull lesion, supraorbital approach, immunohistochemistry

## Abstract

This report describes a rare occurrence of benign fibrous histiocytoma in the frontal sinus of a 38-year-old male. The patient presented with acute symptoms, including sudden-onset headache, nausea, and general discomfort, although neurological, otorhinolaryngological and laboratory examinations showed no abnormalities. A cranial CT scan revealed a cystic, osteodestructive lesion measuring 2.5 cm in the far lateral right frontal sinus, initially suspected to be a mucocele due to radiological characteristics and the patient’s history of recurring frontal headaches and retrobulbar pressure. Elective surgical excision was performed via an external supraorbital approach due to the lesion’s lateral location. Histopathological examination of the excised tissue revealed characteristic features of benign fibrous histiocytoma, including spindle cell proliferation and the presence of histiocytes and siderophages. Immunohistochemistry further supported the diagnosis, showing EMA, S100, and creatinine negativity with SMA positivity. This case is unique, as it represents the first reported benign fibrous histiocytoma in the frontal sinus. During regular follow-up, the patient remained symptom-free and showed no recurrence. This report underscores the importance of considering rare diagnoses for cystic skull lesions and supports tailored surgical approaches based on lesion location.

**Figure 1:**
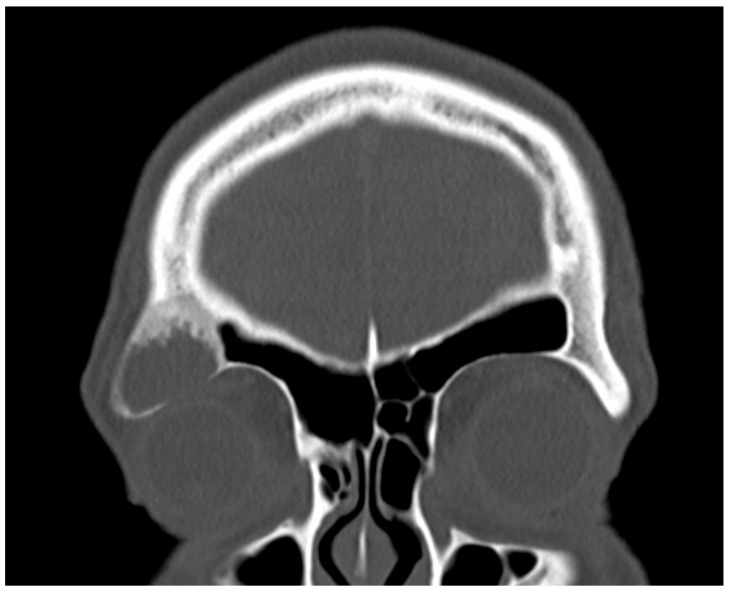
A 38-year-old male presented to the emergency outpatient clinic with sudden-onset headache, nausea, and general discomfort. Neurological and otorhinolaryngological examinations and laboratory analyses revealed no abnormalities. Cranial CT did not indicate any neurovascular events but incidentally detected a cystic, osteodestructive lesion measuring 2.5 cm in diameter in the right frontal sinus. Based on its location, characteristics, and the patient’s history of recurrent frontal headaches and retrobulbar pressure, a mucocele was initially suspected. As the patient’s acute complaints resolved on symptomatic therapy quickly, we decided on elective surgery to drain the plausible mucocele. Due to the lesion’s far lateral position, an endonasal approach was impractical, leading to a supraorbital external approach. A right-sided frontal bone window was created using a piezo device. During the removal of the frontal bone cap, yellowish turbid fluid was discharged from the frontal sinus. The fluid was totally aspirated, and the suspicious mucosa was completely excised and sent for histological analysis. The bony floor of the frontal sinus was found to be thinned and partially eroded, exposing the underlying periorbita, which appeared intact and healthy. The clinical appearance during surgery reinforced the mucocele diagnosis. Subsequently, the bone cap was repositioned, and the external wound was securely closed. The surgery proceeded without any intra- or postoperative complications. Fibrous histiocytoma, or dermatofibroma, is one of the most common cutaneous soft tissue lesions [1]. However, it hardly ever occurs in the skeleton. Its incidence among surgically treated benign skeletal lesions is approximately 1% [2]. The occurrence of benign fibrous histiocytoma in the skull is rarely described in the literature. To our knowledge, this present case is the first confirmed benign fibrous histiocytoma in the frontal sinus. Only one was reported earlier in this location; however, this was a malignant tumor [3]. The first ever reported benign fibrous histiocytoma in the paranasal sinuses was described by Townsend et al. in 1973 [4]. Considering the extremely rare localization and the potential for malignization, the case was presented to the interdisciplinary TUMOR-Board. Since R0 resection is neither clinically nor histologically confirmed and the risk involved in revision surgery is high, close monitoring was chosen, in view of the approximately 11% recurrence rate reported by Bielamowitz et al. [5]. The patient has remained symptom-free without evidence of recurrence, as confirmed by clinical and imaging assessments.

**Figure 2:**
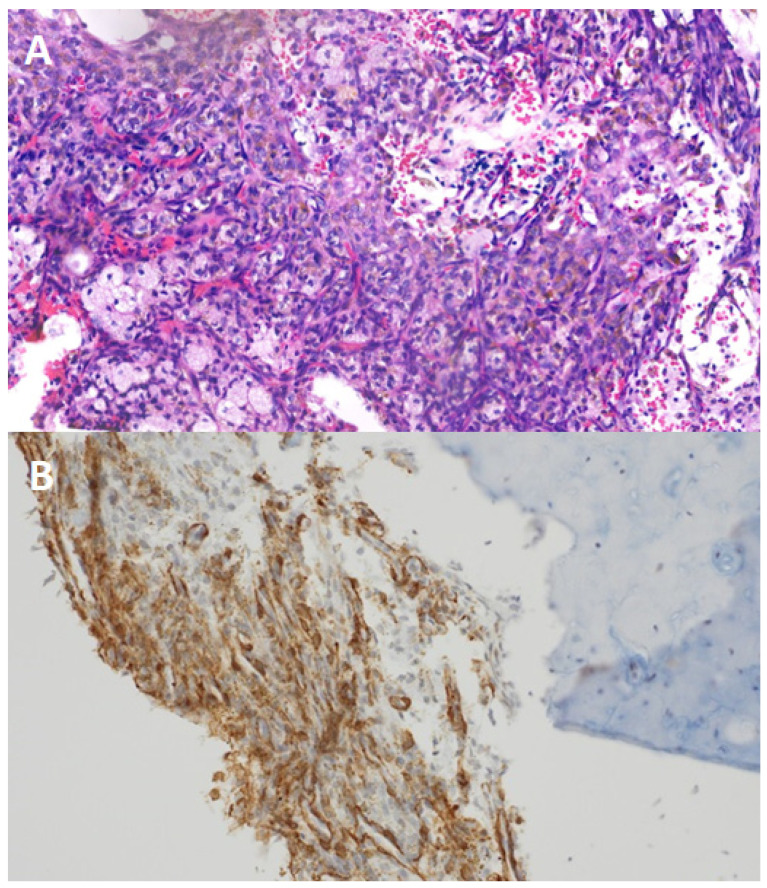
Panel (**A**): histological analysis of the excised lesion revealed histiocytes, siderophages, cholesterol crystals, and spindle cell proliferation without malignancy markers. Panel (**B**): immunohistochemical tests showed EMA, S100, and creatinine negativity but SMA positivity, confirming a benign fibrous histiocytoma.

## Data Availability

No new data were created or analyzed in this study.
